# Regulation of RIP3 by the transcription factor Sp1 and the epigenetic regulator UHRF1 modulates cancer cell necroptosis

**DOI:** 10.1038/cddis.2017.483

**Published:** 2017-10-05

**Authors:** Chengkui Yang, Jun Li, Lu Yu, Zili Zhang, Feng Xu, Lang Jiang, Xiuxia Zhou, Sudan He

**Affiliations:** 1 Cyrus Tang Hematology Center and Collaborative Innovation Center of Hematology, Jiangsu Institute of Hematology, Soochow University, Suzhou, China; 2 Jiangsu Key Laboratory of Preventive and Translational Medicine for Geriatric Diseases, Soochow University, Suzhou, China; 3 Department of Emergency Medicine, First Affiliated Hospital, Soochow University, Suzhou, China

## Abstract

Receptor-interacting kinase-3 (RIP3) is a key regulator of necroptosis. It has been shown that the expression of RIP3 is silenced in most cancer cells and tissues due to genomic methylation. However, the regulatory mechanisms controlling RIP3 expression in cancer cells have not been fully elucidated. Here, we report that Sp1, a well-characterized zinc-finger transcription factor, directly regulates RIP3 expression in cancer cells. Knockdown of endogenous Sp1 significantly decreases the transcription of *Rip*3, thereby further inhibiting necroptosis. The re-expression of Sp1 restores the necroptotic response. In addition, knockdown of epigenetic regulator UHRF1 (ubiquitin-like, containing PHD and RING finger domains 1) in RIP3-null cancer cells reduces the methylation level of the *Rip3* promoter. This effect is sufficient to trigger the expression of RIP3 in RIP3-null cancer cells. The induced expression of RIP3 by UHRF1 RNAi depends on the presence of Sp1. Remarkably, the ectopic expression of RIP3 in RIP3-null cancer cells results in a decrease in tumor growth in mice. Therefore, our findings offer insights into RIP3 expression control in cancer cells and suggest an inhibitory effect of RIP3 on tumorigenesis.

Necrosis is a type of cell death that is morphologically characterized by organelle swelling and plasma membrane rupture. Programmed necrosis or necroptosis has been identified as a form of regulated necrosis that can be induced by a variety of initiators, including death ligands (TNF, TRAIL and Fas),^1, 2^ interferons,^3^ Toll-like receptors (TLRs) ligands^4, 5^ and certain pathogen infections.^6, 7, 8^ Among these, TNF is the most extensively studied inducer for necroptosis. In TNF-induced necroptosis, receptor-interacting kinase (RIP)-1^2, 9^ interacts with RIP3 through the RIP homotypic interaction motif (RHIM) domains of both proteins, leading to the activation of RIP3.^1, 10, 11^ Similarly, the RHIM-containing proteins TRIF, DAI and ICP6, have been shown to activate RIP3 in the necroptosis pathways as induced by, respectively, TLR3/4 ligands,^4^ M45 mutant murine cytomegalovirus^6^ and human herpes simplex virus type 1.^7, 8^ Activated RIP3 phosphorylates the substrate mixed lineage kinase domain-like protein (MLKL).^12, 13^ The phosphorylation of MLKL triggers its oligomerization and plasma membrane localization, eventually leading to the rupture of the cell membrane.^14, 15, 16^ Thus, RIP3 is generally considered to be a central signal-transducing component in the regulation of necroptosis.

The RIP3-dependent necroptosis is involved in many pathological processes, including ischemic injury,^9, 17, 18, 19^ acute inflammatory injury,^20^ neuron degeneration^21, 22^ and inflammatory diseases.^23, 24, 25^ It has been recently reported that the expression of RIP3 in tumor cells and tissues is often silenced due to genetic methylation in the *Rip*3 promoter.^26^ However, the molecular mechanisms underlying the regulation of RIP3 expression in tumor cells have not been fully understood.

Specific protein-1 (Sp1) is a zinc-finger transcription factor that belongs to the Sp/KLF family. Sp1 binds with high affinities to GC-rich promoter elements, including GC-boxes, CACCC-boxes and related motif, which designated as 'Sp1 sites'.^27, 28, 29^ It has been shown that Sp1 regulates the expression of numerous genes involved in cell proliferation, cell cycle and cell death.^30, 31, 32, 33^

In the current study, we demonstrate the requirement of Sp1 in the regulation of RIP3 expression. Knockdown of endogenous Sp1 significantly reduces the transcription of *Rip3* and RIP3-dependent necroptosis. UHRF1 (ubiquitin-like, containing PHD and RING finger domains 1) is a crucial epigenetic regulator in the maintenance of DNA methylation.^34^ We find that downregulation of UHRF1 in RIP3-null cancer cells decreases the methylation level of *Rip3* promoter and further induces the expression of RIP3. This UHRF1 silence-induced RIP3 expression depends on the function of Sp1. Thus, Sp1 and UHFR1 play important roles in the regulation of RIP3 expression and necroptosis in cancer cells. Notably, ectopic expression of RIP3 in cancer cells represses tumor growth in mice, suggesting that lack of RIP3 in most tumor cells facilitates cell survival and tumorigenesis.

## Results

### RIP3 expression sensitizes cancer cells to necroptosis

**Figure 1:**
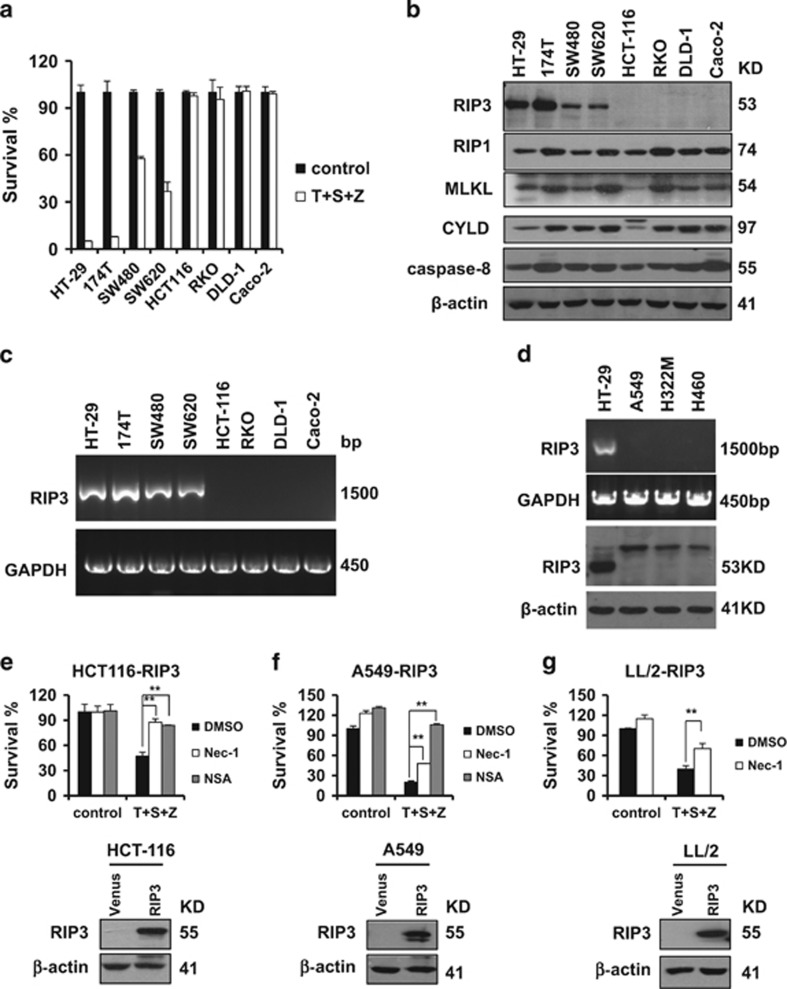
The expression of RIP3 determines the sensitivity of cancer cells to necroptosis. (**a**) The indicated colon cancer cells were treated with DMSO (control) or TNF*α*(T)/Smac mimetic(S)/z-VAD(Z) for 48 h. Cell viability was determined by measuring ATP levels according to the manufacturer’s protocol (CellTiter-Glo Luminescent Cell Viability Assay; Promega). The data are represented as the mean±S.D. of duplicate wells. (**b** and **d**) Western blotting analysis of lysates from the indicated cancer cell lines to measure the protein levels of RIP1, RIP3, MLKL, CYLD, caspase-8 and *β*-actin. (**c** and **d**) Reverse transcription-PCR products from the indicated cells to detect the *Rip3* mRNA. (**e**–**g**) The generated cancer cell lines stably expressing flag-tagged RIP3 were treated with DMSO or T/S/Z plus Nec-1 or NSA for 48 h. Cell viability was determined by measuring ATP levels. The data are represented as the mean±S.D. of duplicate wells. Abbreviations: Nec-1, Necrostatin-1; NSA, Necrosulfonamide

We examined the sensitivity of eight colon cancer cell lines to TNF*α*-induced necroptosis in response to combined treatment with three well-known necroptosis inducers: TNF*α*, Smac mimetic and z-VAD. Four of the cell lines, including 174T, HT-29, SW480 and SW620, responded and underwent TNF*α*-induced necroptosis; the other four lines were resistant to these necroptotic stimuli (Figure 1a). We next measured the expression of necroptotic proteins including RIP3, RIP1, MLKL and CYLD in all of these cell lines. We also monitored the expression of the apoptosis protein caspase-8. Among these, only RIP3 expression correlated with the necroptotic response phenotype in the various cell lines. RIP1, MLKL, CYLD and caspase-8 were ubiquitously expressed in all of the cell lines (Figure 1b). Further, RT-PCR analysis showed that the expression level of *Rip3* mRNA in all of these colon cancer cell lines was correlated with the measured protein levels (Figure 1c). Lack of RIP3 expression was also observed in lung cancer cell lines and these cells were resistant to necroptotic stimuli (Figure 1d and Supplementary Figure S1). Importantly, ectopic RIP3 expression in HCT116 cells made these resistant cells sensitive to TNF-*α* induced necroptotic stimuli (Figure 1e). The observed cell death could be blocked by either RIP1 inhibitor necrostatin-1 or MLKL inhibitor NSA, indicating that HCT116 cells expressing RIP3 were committed to necroptosis upon necroptotic stimuli (Figure 1e). Similar results were observed in both human lung cancer A549 cells and mouse lung cancer LL/2 cells (Figures 1f and g). Taken together, these results suggest that the presence of RIP3 determines the sensitivity of these cancer cells to necroptosis.

### The transcription factor Sp1 regulates *Rip3* transcription

**Figure 2:**
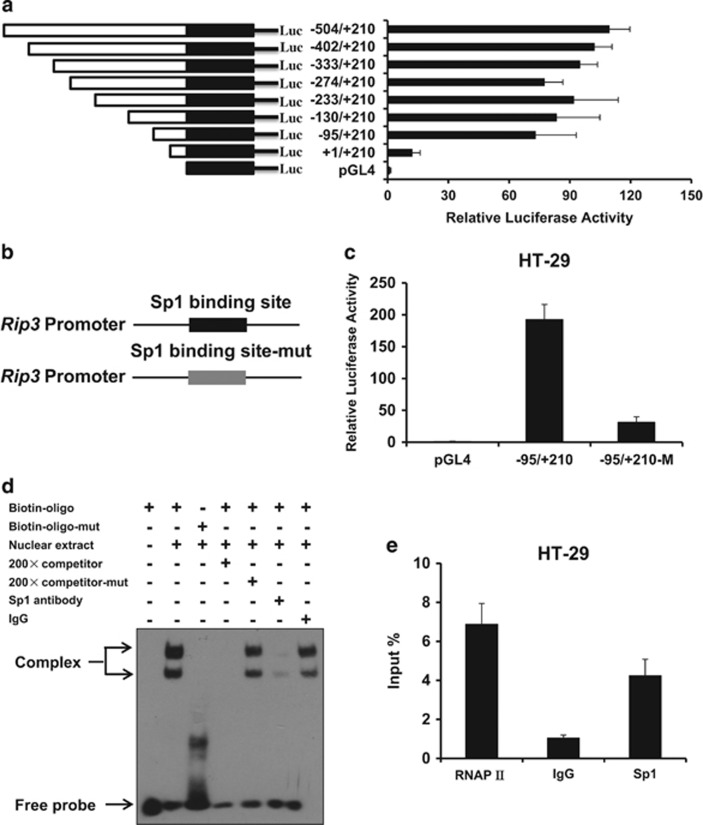
Transcription factor Sp1 regulates RIP3 promoter activity. (**a**) Analysis for transcription activity of *Rip3* promoter in HT-29 cells by the co-transfection of luciferase reporter plasmid (*Rip3*/pGL4 or pGL4) and the pRL-TK plasmid. The luciferase activity value of each sample was first normalized for transfection efficiency by co-transfection with the pRL-TK plasmid. The transcriptional activity of promoter construct was shown as the luciferase activity relative to that of the pGL4 vector (a promoter-less vector). (**b**) Schematic respectively represented WT and site-mutant *Rip3* promoter. (**c**) Analysis for transcription activity of wild type or Sp1 binding site-mutant *Rip3* promoter in HT-29 cells by the co-transfection of luciferase reporter plasmid and the pRL-TK plasmid. (**d**) The binding of transcription factor Sp1 to the *Rip3* promoter was determined by electrophoretic mobility shift analysis (EMSA). By using biotin-labeled 30 bp double-stranded oligonucleotides containing wild type or mutated Sp1 binding sites as probes, EMSAs were performed with the same amount of nuclear extracts (10 *μ*g) from HT-29 cells, and the products were separated on a 6% polyacrylamide gel (lanes 2–7). Lane 1, free probe; lane 2, biotin-labeled wild-type Sp1 consensus oligonucleotides were mixed with nuclear proteins; lanes 3, binding assays of biotin-labeled mutant-type Sp1 consensus oligonucleotides mixed with nuclear proteins; lane 4, the same reaction was performed as that in lane 2, except for the presence of a 200-fold excess of unlabeled wild-type Sp1 consensus oligonucleotides as a competitor; lane 5, the same reaction was performed as that in lane 2, except for the presence of a 200-fold excess of unlabeled mutant-type Sp1 consensus oligonucleotides as a competitor; lanes 6–7, IgG or anti-Sp1 antibody was added to the binding reaction mixtures with biotin-labeled wild-type probe. (**e**) ChIP assay using antibody against Sp1 was performed in HT-29 cells. The normal rabbit IgG was used as a negative control, the anti-polymerase-II antibody was used as a positive control and input indicates 5% input DNA, a positive amplification control

To investigate the mechanism governing the expression of RIP3, we first examined the transcription activity of *Rip3* promoter in HT-29 cells using luciferase reporter assay. We generated eight luciferase constructs harboring different length DNA fragments of the candidate *Rip3* promoter. As shown in Figure 2a, the region from −95 bp to +210 bp had strong promoter activity in HT-29 cells. Using the sequence of this region as a query, we searched for putative transcription factor binding sites by using ALGGEN-PROMO software (Supplementary Figure S2). One of the top hits was the zinc-finger transcription factor Sp1, which was predicted to bind directly to putative binding site located at −4 and 4 in the *Rip3* promoter. We evaluated the impact of Sp1 on *Rip3* promoter activity by generating mutations that disrupted the Sp1 binding site. Transcription activity of *Rip3* promoter was significantly reduced when *Rip3* promoter lost its ability to bind Sp1 (Figures 2b and c). Further, we performed electrophoretic mobility shift assay (EMSA) with biotin-labeled oligonucleotides containing the Sp1 binding site as the wild-type probe. Super shifted complex was detected in the presence of the biotin-labeled probe (Biotin-oligo) (Figure 2d, lane 2), while this super shifted complex disappeared when the 200-fold free unlabeled probe (competitor) was added to the reaction (Figure 2d, lane 4). In order to clarify whether the Sp1 binding site in *Rip3* promoter is required for the observed supershift complex, we performed EMSA with biotin-labeled oligonucleotides containing mutations in the Sp1 binding site (Biotin-oligo-mut) as the mutant probe. Mutation of the Sp1 binding site dramatically decreased the formation of super shifted complex (Figure 2d, lane 3). Moreover, 200-fold free unlabeled mutant probe was unable to compete with biotin-labeled wild-type probe to destroy the formation of super shifted complex (Figure 2d, lane 5). To step further confirm the specific binding of Sp1 to the *Rip3* minimal promoter region, EMSA was performed with anti-Sp1 antibody. Super shifted complex was observed in the presence of IgG, while no-shift was detected when anti-Sp1 antibody was added (Figure 2d, lane 6 and lane 7). To further demonstrate the interaction between transcription factor Sp1 and *Rip3* promoter, we performed chromatin immunoprecipitation (ChIP) assay in HT-29 cells. A strong Sp1 binding activity to the *Rip3* promoter was observed (Figure 2e). These suggest that transcription factor Sp1 specifically binds to *Rip3* promoter and regulates *Rip3* transcription.

### Sp1 modulates RIP3-mediates necroptosis

**Figure 3:**
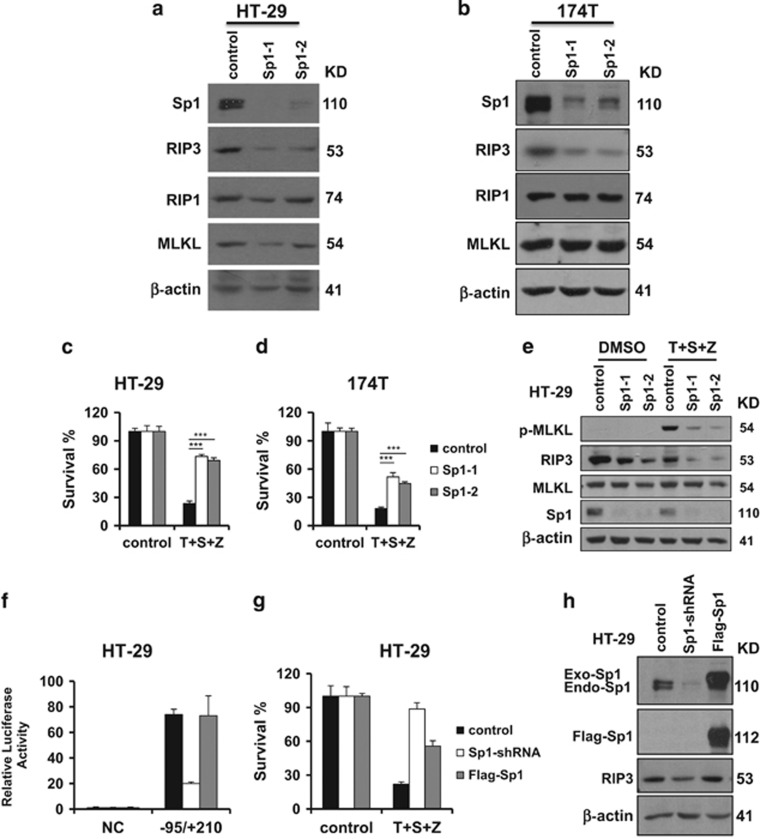
Sp1 regulates the expression of RIP3 and necroptosis. (**a** and **b**) HT-29 or 174T cells stably expressing Sp1-shRNA or control vector were generated as described in the Materials and Methods. Western blotting analysis of lysates from the indicated stable cell lines showing Sp1, RIP1, RIP3, MLKL and *β*-actin levels. (**c** and **d**) The indicated control or Sp1-shRNA stable cell lines were treated with DMSO or T/S/Z for 24 h. Cell viability was determined by measuring ATP levels. ***P<0.001. (**e**) The indicated control or Sp1-shRNA stable cell lines were treated with DMSO or T/S/Z for 8 h. Cell lysates were collected and aliquots of 40 *μ*g were subjected to western blot analysis of p-MLKL, RIP3, MLKL, Sp1 and *β*-actin levels. (**f**) Analysis for transcription activity of *Rip3* promoter by the co-transfection of luciferase reporter plasmid in HT-29 cells, Sp1-shRNA and WT-Sp1 cell lines. Sp1-shRNA: HT-29 cells stably expressing an shRNA targeting Sp1. Flag-Sp1: Sp1-shRNA cells stably expressing an shRNA-resistant Sp1. (**g**) HT-29 cells, Sp1-shRNA and Flag-Sp1 cell lines were treated as indicated for 24 h. Cell viability was determined by measuring ATP levels. (**h**) Western blotting analysis of lysates from the indicated cell lines showing Sp1, RIP3 and *β*-actin levels

To examine the biological function of Sp1 in RIP3-mediated necroptosis, we stably knocked down Sp1 expression in HT-29 cells and 174T cells using the RNAi approach. Knockdown of Sp1 dramatically decreased the expression level of RIP3 in these cells (Figures 3a and b). Consistently, decreased Sp1 binding activity to *Rip3* promoter was observed in Sp1-shRNA HT-29 cells by CHIP assay (Supplementary Figure S3). Notably, stable knockdown of Sp1 in either HT-29 cells or 174T cells blocked TNF*α*-induced necroptosis (Figures 3c and d). Moreover, the level of phosphorylated MLKL was significantly reduced in Sp1-shRNA cells (Figure 3e). In Sp1-shRNA cells, re-expression of an shRNA-resistant Sp1 transgene rescued *Rip3* promoter activity (Figure 3f) and TNF*α*-induced necroptosis (Figures 3g and h). As Sp1 has been shown to activate TNF*α* transcription,^35, 36^ we investigate the role of Sp1 in TNF*α* production in the cells treated with TNF/Smac mimetic/z-VAD. TNF*α* mRNA was induced by TNF/Smac mimetic/z-VAD in HCT116-RIP3 cells (Supplementary Figure S5). However, this induction was not inhibited by the knockdown of Sp1 in HCT116-RIP3 cells (Supplementary Figure S5). Taken together, these results suggest that Sp1 is able to modulate necroptosis by regulating RIP3 expression.

### The epigenetic regulator UHRF1 controls epigenetic silencing of RIP3 in cancer cells

**Figure 4:**
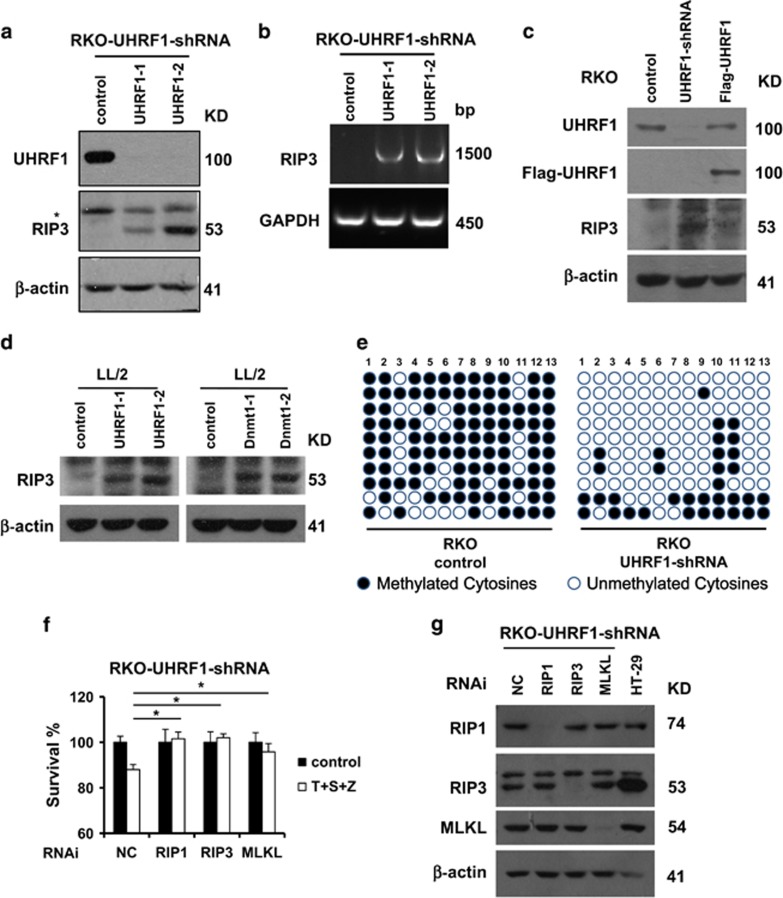
The epigenetic regulator UHRF1 controls epigenetic silencing of RIP3 in cancer cells**.** (**a**) RKO cells, UHRF1-shRNA stable cell lines were generated as described in the Materials and Methods, and western blotting analysis of lysates from the indicated stable cell lines showing UHRF1, RIP3 and *β*-actin levels. RKO-UHRF1-shRNA: RKO cells stably expressing a shRNA targeting UHRF1. (**b**) Reverse transcription-PCR products from the indicate cells to detect the *Rip3* mRNA. (**c**) Western blotting analysis of lysates from the indicated stable cell lines showing UHRF1, Flag, RIP3 and *β*-actin levels. Flag-UHRF1: UHRF1-shRNA cells transiently expressing an shRNA-resistant UHRF1. (**d**) RNAi of UHRF1 and Dnmt1 in LL/2 cells. Western blotting analysis of lysates from the indicated stable cell lines showing RIP3 and *β*-actin levels. (**e**) Methylation status of the CpG-dinucleotides of DNA sequences (−152 to +240 bp) upstream and downstream of RIP3 transcription start site was validated by bisulfite sequencing from the indicated cell lines. (**f**) RKO-UHRF1-shRNA cells were transfected with the control, RIP1 siRNA, RIP3 siRNA or MLKL siRNA. Forty-eight hours post transfection, cells were treated as indicated for additional 48 h. Cell viability was determined by measuring ATP levels. *P<0.05. (**g**) The knockdown efficiency of RIP1, RIP3 and MLKL RNAi. Cell lysates were collected 48 h post transfection and aliquots of 40 *μ*g were subjected to western blot analysis of RIP1, RIP3, MLKL and *β*-actin levels. All experiments were repeated at least three times with similar results

As it has been recently reported that DNA methylation silenced the expression of RIP3, we analyzed the methylation status of *Rip3* promotor in both RIP3-positive and RIP3-negative cells. Hypomethylation of *Rip3* promotor was observed in RIP3-positive HT-29 cells, while RIP3-negative HCT116 cells showed highly methylation status of *Rip3* promotor (Supplementary Figure S6). We then examined the role of UHRF1 in the regulation of RIP3 expression. As shown in Figures 4a and b, UHRF1 knockdown in RKO cells induced RIP3 expression both at mRNA and protein levels. Moreover, reconstitution of UHFR1 in UHRF1-shRNA cells reduced RIP3 expression (Figure 4c). Unexpectedly, knockdown of DNA methyltransferase 1 (Dnmt1) was unable to induce RIP3 expression in RKO cells (Supplementary Figures S7a and b). In the mouse LL/2 cells, knockdown of either UHRF1 or Dnmt1 could induce RIP3 expression (Figure 4d and Supplementary Figure S7c). To examine the correlation between DNA methylation and the expression level of RIP3, we further investigated the methylation status of *Rip3* promoter region in UHRF1-shRNA RKO cells by bisulfite sequencing PCR sequencing. RKO cells expressing control-shRNA exhibited a denser methylation pattern at *Rip3* promoter region compared with UHRF1-shRNA cells (Figure 4e). Furthermore, the induced expression of RIP3 could sensitize RKO cells to TNF*α*-induced necroptosis (Figures 4f and g). Consistently, this cell death was blocked by knockdown of RIP3, RIP1 or MLKL (Figures 4f and g). These findings suggest that UHRF1 is essential for the maintenance of hypermethylation of *Rip3* promoter and thus contributes to the silenced RIP3 expression in cancer cell lines.

### Downregulation of UHRF1 promotes endogenous RIP3 expression in an Sp1-dependent manner

**Figure 5:**
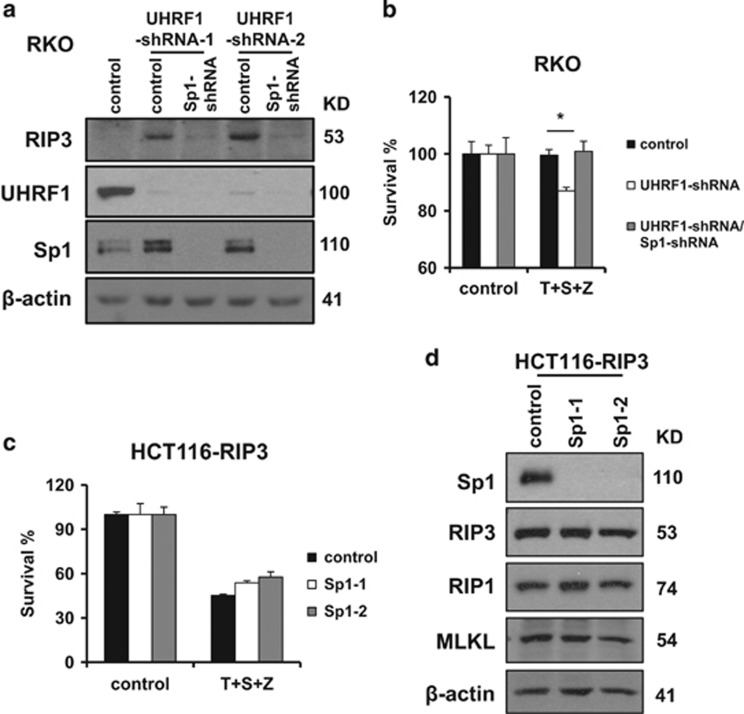
Downregulation of UHRF1 promotes RIP3 expression depending on Sp1. (**a**) RKO stably expressing UHRF1-shRNA and Sp1-shRNA cells were generated as described in the Materials and Methods, and western blotting analysis of lysates from the indicated cell lines showing RIP3, UHRF1, Sp1 and *β*-actin levels. (**b**) The indicated cells lines were treated as indicated for 48 h. Cell viability was determined by measuring ATP levels. *P<0.05. (**c**) HCT116-RIP3 stably expressing Sp1-shRNA cells were generated as described in the Materials and Methodds. The indicated cell lines were treated as indicated for 48 h. Cell viability was determined by measuring ATP levels. (**d**) Western blotting analysis of lysates from the indicated cells were showing Sp1, RIP1, RIP3, MLKL and *β*-actin levels

To illustrate whether Sp1 is required for the induced RIP3 expression by UHRF1 knockdown, we stably knock down the expression of Sp1 in UHRF1-shRNA RKO cells. Sp1 knockdown greatly decreased the expression level of RIP3 in UHRF1-shRNA RKO cells (Figure 5a). This result indicates that Sp1 is critical for *Rip3* transcription in response to downregulation of UHRF1. Moreover, knockdown of Sp1 blocked TNF-induced necroptosis by inhibiting the induction of *Rip3* in UHRF1-shRNA RKO cells (Figure 5b). These results suggest that Sp1 plays an important role in promoting *Rip3* transcription in response to decreased methylation level of *Rip3* promoter in *Rip3*-null cancer cells. We further evaluated the effect of Sp1 on ectopically expressed RIP3 in HCT116 cells. As expected, knockdown of Sp1 had no impact on the expression level of exogenous RIP3 and TNF-induced necroptosis in HCT116 cells ectopically expressing RIP3 (Figures 5c and d). This result further supports the important role of Sp1 in the regulation of *Rip3* transcription.

### Expression of RIP3 in cancer cells represses tumor growth in mouse

**Figure 6:**
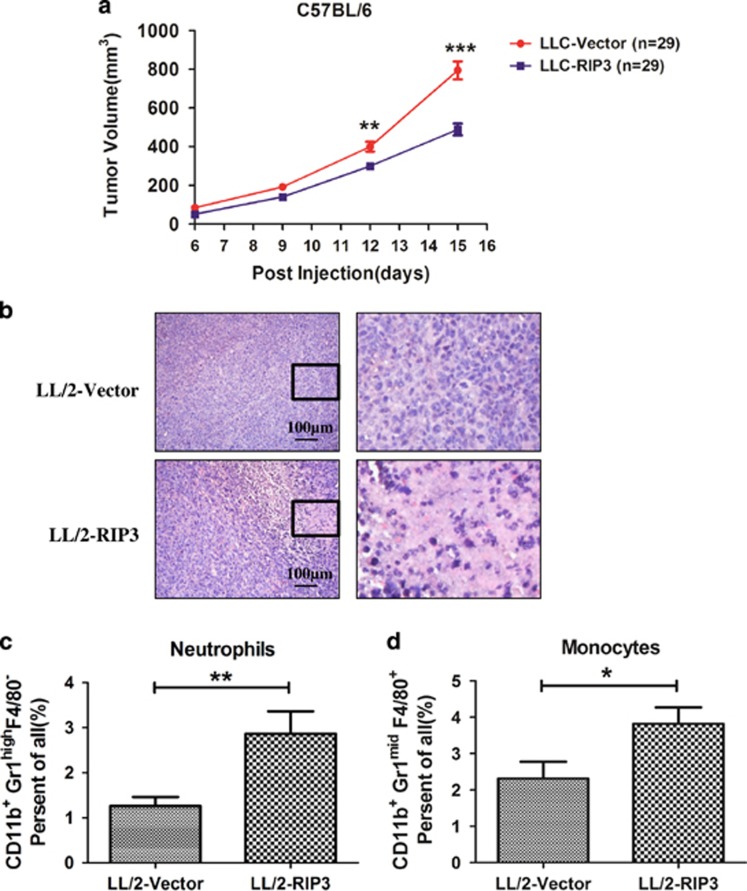
Expression of RIP3 in cancer cells represses tumor growth in mouse. (**a**) Effects of RIP3 overexpression in LL/2 lung cancer cells xenograft in C57BL/6 mice. Tumor volume was monitored and shown in the graph. Data are presented as means±S.D. (*n*=29). (**b**) Parts of the tumor issue were fixed and embedded in paraffin, followed by hematoxylin/eosin staining. (**c** and **d**) Infiltration of monocytes and neutrophils into the tumor tissues was analyzed by flow cytometry analysis

To functionally analysis the potential role of RIP3 in tumor growth, we generated stable RIP3-expressing LL/2 lung cancer cells and these cells had the ability to undergo necroptosis (Figure 1g). The generated LL/2 cells expressing RIP3 (LL/2-RIP3) or vector (LL/2-Vector) had equal proliferation rates (Supplementary Figure S4). These two cell lines were individually injected into immunocompetent C57BL/6 mice. Tumors derived from LL/2-RIP3 cells showed a significantly decreased tumor volume compared with those from LL/2-Vector cells (Figure 6a). The exogenous RIP3 was still expressed in LL2/RIP3 xenografts (Supplementary Figure S8b). Interestingly, increased level of cell death was observed in the tumor sections from LL/2-RIP3 tumors compared with that in LL/2-Vector tumors (Figure 6b and Supplementary Figure S8a). Moreover, the infiltration of monocytes and neutrophils into tumor sections was significantly increased in LL/2-RIP3 tumor (Figures 6c and d), which may contribute to tumor suppression. These results implicate that expression of RIP3 in cancer cells represses tumor growth in mice.

## Discussion

**Figure 7:**
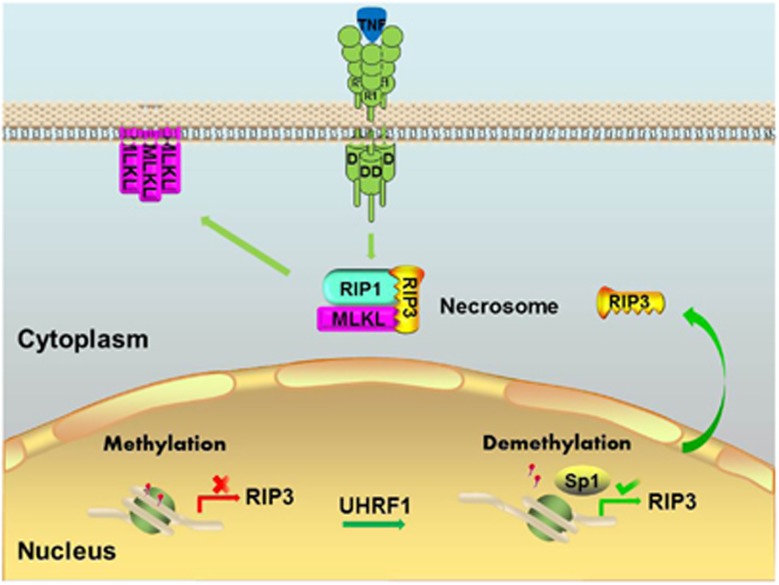
Regulation of RIP3 expression and necroptosis by UHRF1 and Sp1 in cancer cells

Necroptosis has been shown to play important roles in various pathophysiological conditions, including ischemic injuries,^9, 17, 18, 19^ inflammatory diseases^20^ and neurodegeneration.^21, 22^ RIP3 is emerging as a key molecule in the regulation of necroptosis.^1, 10, 11^ Interestingly, most cancer cells display a defect in RIP3, thereby obtaining the ability to evade necroptosis.^26^ In the current study, we demonstrate the roles of transcript factor Sp1 and epigenetic regulator in the regulation of RIP3 expression in cancer cells (Figure 7). In most cancer cells, *Rip3* promoter is methylated due to aberrant DNA methylation patterns, resulting in silence of RIP3. The defective necroptosis in RIP3-null cancer cells contributes to the growth and survival of cancer cells. Knockdown of UHRFI is sufficient to reduce methylation level of *Rip3* promoter. This process allows Sp1 to promote *Rip3* transcription. The induced expression of RIP3 protein sensitizes cancer cells to necroptosis, leading to inhibition of tumor growth.

Gene transcription is tightly regulated by specific transcription factor(s). However, the essential transcription factor for *Rip3* is uncharacterized. Here, we identified Sp1 as an important transcript factor in the regulation of *Rip3* through directly binding to the promoter region near the *Rip3* transcription start site. Stable knockdown of Sp1 significantly reduced the expression of RIP3 in both HT-29 and 174T cells (Figures 3a and b). It is known that Sp1 regulates the transcription of a variety of genes. We further examined the expression levels of other necroptosis regulators RIP1 and MLKL. Although we have observed dramatic reduction of RIP3 level in Sp1-shRNA cells, no obvious differences in RIP1 and MLKL levels were detected between control-shRNA cells and Sp1-shRNA cells (Figures 3a and b). These results suggest that Sp1 regulates the transcription of *Rip3* rather than *Rip1* and *Mlkl*. It is important to note that knockdown of Sp1 inhibits TNF-induced necroptosis and this defect is rescued by re-expression of Sp1. Our results demonstrate that Sp1 is required for RIP3 expression and RIP3-mediated necroptosis.

DNA methylation is an epigenetic modification that generally leads to the suppression of gene expression. Aberrant DNA methylation patterns are commonly observed in many cancers.^37^ UHRF1 plays an essential role in the maintenance of DNA methylation.^34^ UHRF1 recruits DNA methyltransferase 1 (Dnmt1) to the hemimethylated DNA to ensure efficient maintenance of methylation patterns.^34^ In RIP3-null RKO cells, knockdown of UHRF1 reduced the methylation level of *Rip3* promoter and induced the expression of both RIP3 mRNA and protein, while knockdown of Dnmt1 failed to induce RIP3. We also found that knockdown of either UHRF1 or Dnmt1 in the mouse LL/2 cells induced RIP3 expression (Figure 4d and Supplementary Figure S7c). This result is consistent with previous observation that Dnmt1 knockdown led to the induction of RIP3.^26^ The distinct impact of Dnmt1 knockdown on RIP3 expression might be due to cell-type-dependent differences. Since knockdown of UHRF1 in RKO cells reduced the methylation level of *Rip3* promotor, we further analyzed the expression levels of UHRF1 and Sp1 between RIP3-expressing and null cells. As shown in Supplementary Figure S9, there was no obvious difference in the expression levels of UHRF1 and Sp1 between RIP3-expressing and null cells. It is tempting to speculate that there might be additional molecules acting upstream of UHRF1 to determine the methylation level of *Rip3* promotor and RIP3 expression level among the cancer cells.

Recently, RIP3 has been implicated in the suppression of myeloid leukemogenesis by promoting cell death and differentiation of leukemia-initiating cells.^38^ We investigate the role of RIP3 on tumorigenesis in a lung cancer model. Stable expression of RIP3 LL/2 lung cancer cells renders cells sensitive to necroptotic inducer (Figure 1g). Notably, ectopic expression of RIP3 results in significantly reduced *in vivo* tumor growth (Figure 6a). In the tumor tissue, elevated necrotic cell death and infiltration of monocytes and neutrophils were detected in LL/2 cells expressing RIP3 compared with cells expressing control vector (Figures 6b–d). These results suggest that defective expression of RIP3 facilitates cancer cells survival and tumor growth. In summary, our findings provide new insights into the regulatory mechanism of RIP3 and evidence for a tumor-suppressive role of RIP3 in cancer cells. The demethylating drugs such as decitabine have been used for the treatment of myelodysplastic syndrome,^39^ acute myeloid leukemia^40^ and is also being investigated for use in many tumors. The induced RIP3 and necroptotic response might be one of the mechanisms that benefit patients treated with the demethylating drug.

## Materials and methods

### Reagents and antibodies

TNF-*α* recombinant protein was generated as previously described.^1^ z-VAD was from Bachem (Babendorf, Switzerland). Necrostatin-1 was purchased from Alexis Biochemicals (San Diego, CA, USA). The following antibodies were used for western blot analysis: RIP1 (BD Biosciences, NJ, USA, 610458), phospho-MLKL (Abcam, Cambridge, UK, 187091), caspase-8 (Cell Signaling, Danfoss, MA, USA, 9746), CYLD (Cell Signaling, 437700), mouse RIP3 (Prosci (San Diego, CA, USA), 2283), Sp1 (Santa Cruz, CA, USA, 14027), UHRF1 (BD Biosciences, 612264), Dnmt1 (Abcam, 13537), Flag (Sigma, St. Louis, MO, USA, A8592) and *β*-actin (Sigma, A2066). The following antibodies were used for flow cytometry analysis: CD11b (Biolegend, San Diego, CA, USA, 101212), CD45 (BD, 563891), GR-1 (BD Biosciences RB6-8C5), F4/80 (Biolegend, 123113).

### Cell culture

HT-29 cells were maintained in McCoy's 5A culture medium. 174T, SW480, SW620, HCT116, RKO, DLD-1, Caco-2, A549, H322M, H460 and LL/2 were cultured in DMEM medium. All media contains 10% fetal bovine serum (Invitrogen, CA, USA) and 100 units/ml penicillin–streptomycin (Hyclone, Logan, UT, USA).

### Plasmids and RNA interference

For the RIP3 and Sp1 lentivirus expression constructs, mouse RIP3, human RIP3 and human Sp1 were cloned into the pCDH vector. The *Rip3* promoter was amplified by PCR from genomic DNA in HT-29 cells and cloned into pGL4 vector. The expression construct containing Sp1 binding site mutant of *Rip3* promoter was generated by using mutagenesis kit (Stratagene, La Jolla, CA, USA). RIP1 siRNA (5′-gaaagaguauucaaacgaa-3′), RIP3 siRNA (5′-ccagagaccucaacuuuca-3′) and MLKL siRNA (5′-gaaagaguauucaaacgaa-3′) were synthesized by Dharmacon (CO, USA). RNA interference was performed using lipofectamine 2000 reagent (Invitrogen). To generate lentiviral particles, the following shRNA oligonucleotides (Sigma) were cloned into pLKO.1 vector: UHRF1 (TRCN0000273256 and TRCN0000273313), Dnmt1 (TRCN0000021890 and TRCN0000021893) and Sp1 (TRCN0000020448 and TRCN0000274208).

### Lentivirus expression system

For producing lentiviral particles, pCDH plasmid containing the target gene (mouse RIP3, human RIP3, human Sp1), ΔR, VSVG and Rev were co-transfected into 293T cells. Twelve hours later, the media was changed to remove the transfection reagents and replaced with 5 ml fresh complete media. The supernatants were harvested 48–72 h post transfection. To generate HCT116, A549 and LL/2 cancer cells stably expressing RIP3, the indicated cells were infected with 1 ml of supernatant lentiviral particles expressing RIP3. HT-29 cells are infected with 1 ml of supernatant lentiviral particles expressing Sp1 to generate stably expressing Sp1. Seventy-two hours post infection, the indicated cells were sorted by a flow cytometer (BD Biosciences, NJ, USA) for GFP fluorescence and finally used as stable cells. For another lentiviral particles, pLKO.1 plasmid harboring shRNA oligonucleotides together with psPAX2 and pMD2.G were co-transfected into 293T cells. The supernatant were collected 48 h post tranfection, and then were used to infect cultured cells for 48 h. The culture media were replaced with fresh puromycin containing media every 2 days. After around 10 days, knockdown efficiency was assessed using western blotting. An empty pLKO.1 vector was used for the generation of control cell line. To generate Sp1-shRNA-resistant scramble expression constructs, we mutated six nucleotides within the shRNA targeting region on Sp1 without affecting amino acid sequence.

### Reverse transcription-PCR

Total RNA was extracted using TRIzol reagent (Invitrogen). Reverse transcription was carried out using the PrimeScript RT Master Mix kit (Takara, Dalian, China). The PCR products were resolved using an agarose gel containing GelRed Nucleic Acid Gel Stain (Biotium, Fremont, CA, USA), and exposed on a Kodak Image Station 440CF (Kodak, Rochester, NY, USA).

### Protein extraction and western blotting

Cell pellet was collected and re-suspended in protein lysis buffer. Cell lysate was then incubated on ice for 20 min, and centrifuged at 13 000 × *g* for 20 min. The supernatants were collected for western blot analysis. Equal amounts (40 *μ*g) of total protein were loaded, and then subsequently immunoblotted with the primary antibodies.

### 5-Aza-2-deoxycytidine treatment

5-Aza-2-deoxycytidine (Sigma, A3656) was prepared as a 20 mM stock solution in 50% acetic acid protected from light. 5-Aza-2-deoxycytidine was used at 1–25 *μ*M concentrations for 5 days treatment durations as detailed in figure legends. All mediums were daily changed. Finally, genomic DNA and protein were extracted from treated cells for PCR and western blot analyses.

### Transfection and luciferase reporter assay

HT-29 cells were co-transfected with pGL4 containing the candidate *Rip3* promoter sequence and RL-TK plasmids by using lipofectamine 2000 reagent (Invitrogen). Cells were harvested to determine luciferase activity 48 h post transfection using the Dual-Luciferase Reporter Assay System kit (Promega, WI, USA, E1980) by a luminometer instrument (Thermo Scientific, MA, USA). All experiments were conducted in triplicate.

### Genomic DNA extraction and bisulfite sequencing PCR

Genomic DNA was extracted from cultured cells using TIANamp Genomic DNA Kit (Tiangen, Beijing, China), DP304. CpG island region in Rip3 promoter was amplified with sodium bisulfite-treated DNA as a template using EpiTect Bisulfite kit (Qiagen, Shanghai, China, 59104) under the following PCR conditions: 95 °C for 5 min, and then 60 °C for 25 min, followed by 95 °C for 5 min, 60 °C for 85 min, 95 °C for 5 min, 60 °C for 175 min and ended by a final step at 20 °C overnight. The DNA were eluted and used as a template for PCR amplification. The purified PCR products were cloned into the pGEM-T easy vector (Promega), and 10 clones at least for each sample sequenced by Genewiz Co. (Suzhou, China).

### ChIP assay

ChIP assay was performed using the ChIP kit (Millipore, MA, USA, 17-371RF) according to the manufacturer’s protocol. Briefly, HT-29 cells were crosslinked with formaldehyde (1%) for 10 min at room temperature followed by quenching with glycine for 5 min. Fixed cells were washed with PBS and then suspended in SDS lysis buffer. Cell lysates were further incubated with 1.5 *μ*g of anti-Sp1 antibody or IgG at 4 °C overnight. The immunoprecipitated DNA was amplified by the promoter-specific primers.

### Electrophoretic mobility shift assays

EMSA was performed by using the LightShift Chemiluminescent EMSA Kit (Thermo Scientific, 20148X) according to the manufacturer’s protocol. Briefly, the DNA binding reaction was carried out in a 20 *μ*l reaction mixture containing 10 *μ*g HT-29 nuclear extracts, and the wild-type biotin-labeled probe or mutated biotin-labeled probe, with or without 200-fold biotin-unlabeled competitor. For supershift reactions, nuclear extracts were pre-incubated with 1.5 *μ*g anti-Sp1 or IgG antibody on ice for 1 h. This reaction was then subjected to none-reduced PAGE and transferred to a nylon membrane. The biotin-labeled DNA was incubated with Streptavidin-Horseradish Peroxidase Conjugate and detected with Chemiluminescent Substrate.

### Tumor models

We used female C57BL/5 mice at the age of 6–8 weeks. LL/2 cells (0.2 million cells) in 50 *μ*l PBS were injected subcutaneously into the right buttock area of each mouse. After 15 days, mice were killed and the tumor tissues were collected for further analysis. We selected at least three tumor areas within the same tumor at random and calculated the proportion of the necrotic area on the whole sample area.

### Flow cytometry analysis

Tumor tissues were harvested, minced and digested at 37 °C for 40 min with DMEM containing hyaluronidase (1.5 mg/ml), collagenase type 1A (1.5 mg/ml) and DNase (2 mg/ml). The digestion mixtures were filtered through 70-*μ*m cell strainers, and then were stained by related antibodies in cold buffer (1% BSA, 0.1% NaN_3_ in PBS). Flow cytometry data were acquired on a Gallios flow cytometer (Beckman Coulter, Brea, CA, USA) and were analyzed with Kaluza for Gallios software (Beckman Coulter).

## Publisher’s Note

Springer Nature remains neutral with regard to jurisdictional claims in published maps and institutional affiliations.
