# Supplementary material for: Regulation of RIP3 by the transcription factor Sp1 and the epigenetic regulator UHRF1 modulates cancer cell necroptosis
Source: Cell Death Dis. 2017 Oct 5;8(10):e3084–. doi: 10.1038/cddis.2017.483 (PMC5682651; doi:10.1038/cddis.2017.483)
Supplement: Supplementary Figure S1 [file cddis2017483x1.ppt]

## Slide 1
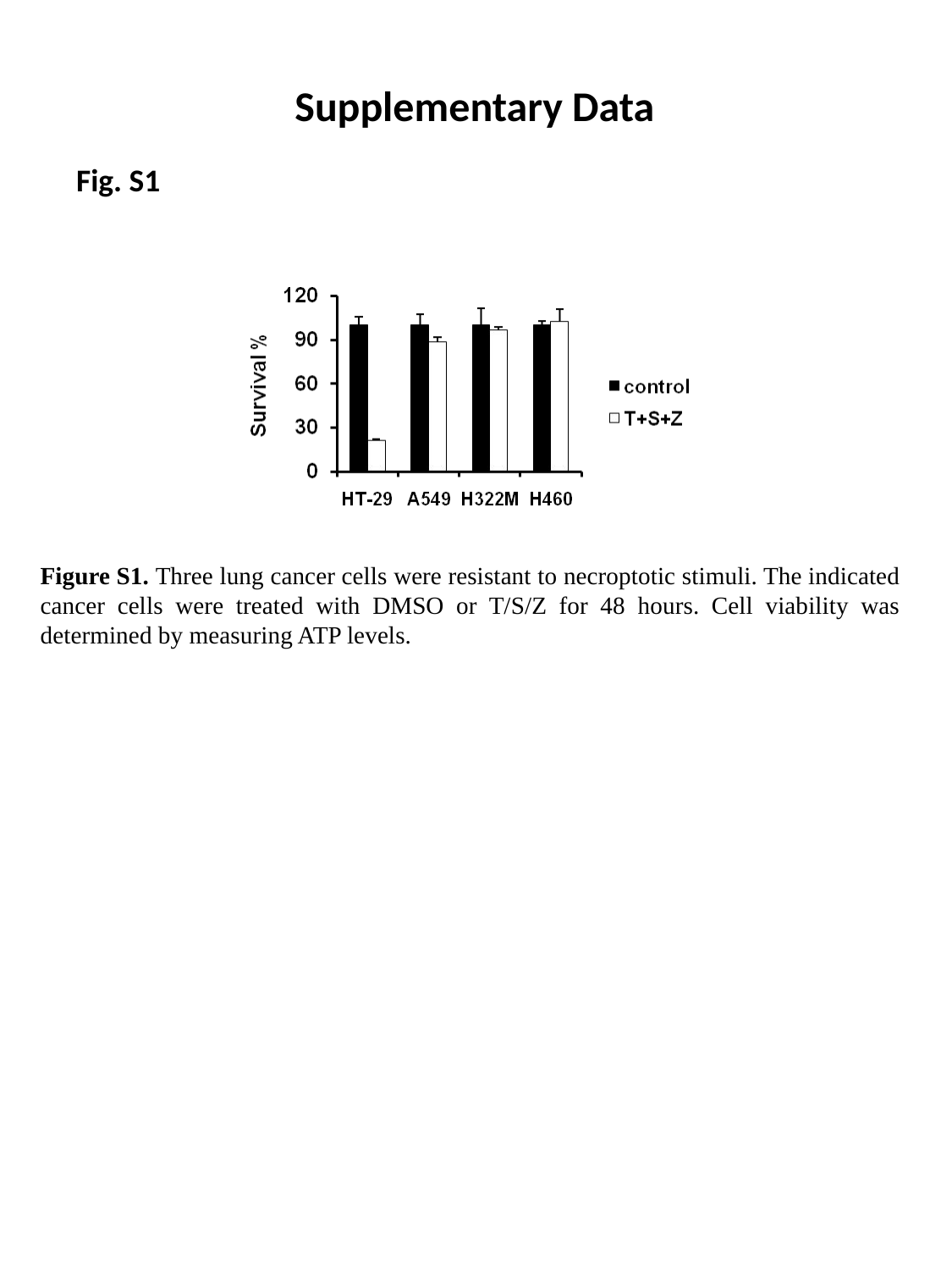

Supplementary Data
Fig. S1
Figure S1. Three lung cancer cells were resistant to necroptotic stimuli. The indicated cancer cells were treated with DMSO or T/S/Z for 48 hours. Cell viability was determined by measuring ATP levels.
