# Supplementary material for: Regulation of RIP3 by the transcription factor Sp1 and the epigenetic regulator UHRF1 modulates cancer cell necroptosis
Source: Cell Death Dis. 2017 Oct 5;8(10):e3084–. doi: 10.1038/cddis.2017.483 (PMC5682651; doi:10.1038/cddis.2017.483)
Supplement: Supplementary Figure S2 [file cddis2017483x2.ppt]

## Slide 1
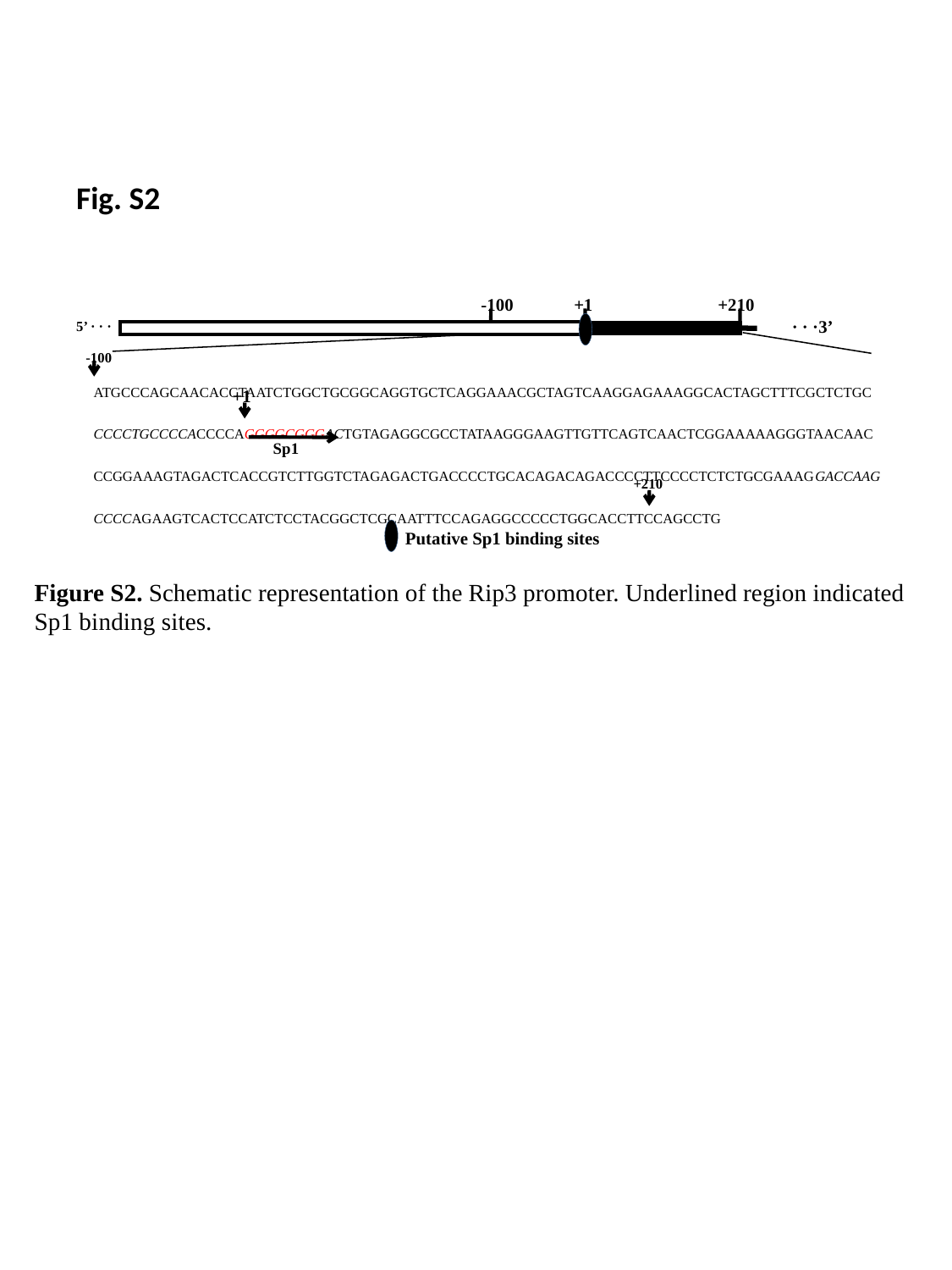

Fig. S2
-100
+1
+210
· · ·3’
5’ · · ·
-100
ATGCCCAGCAACACCTAATCTGGCTGCGGCAGGTGCTCAGGAAACGCTAGTCAAGGAGAAAGGCACTAGCTTTCGCTCTGCCCCCTGCCCCACCCCAGGGGCGGGACTGTAGAGGCGCCTATAAGGGAAGTTGTTCAGTCAACTCGGAAAAAGGGTAACAACCCGGAAAGTAGACTCACCGTCTTGGTCTAGAGACTGACCCCTGCACAGACAGACCCCTTCCCCTCTCTGCGAAAGGACCAAGCCCCAGAAGTCACTCCATCTCCTACGGCTCGCAATTTCCAGAGGCCCCCTGGCACCTTCCAGCCTG
+1
Sp1
+210
Putative Sp1 binding sites
Figure S2. Schematic representation of the Rip3 promoter. Underlined region indicated Sp1 binding sites.
