# Supplementary material for: Regulation of RIP3 by the transcription factor Sp1 and the epigenetic regulator UHRF1 modulates cancer cell necroptosis
Source: Cell Death Dis. 2017 Oct 5;8(10):e3084–. doi: 10.1038/cddis.2017.483 (PMC5682651; doi:10.1038/cddis.2017.483)
Supplement: Supplementary Figure S3 [file cddis2017483x3.ppt]

## Slide 1
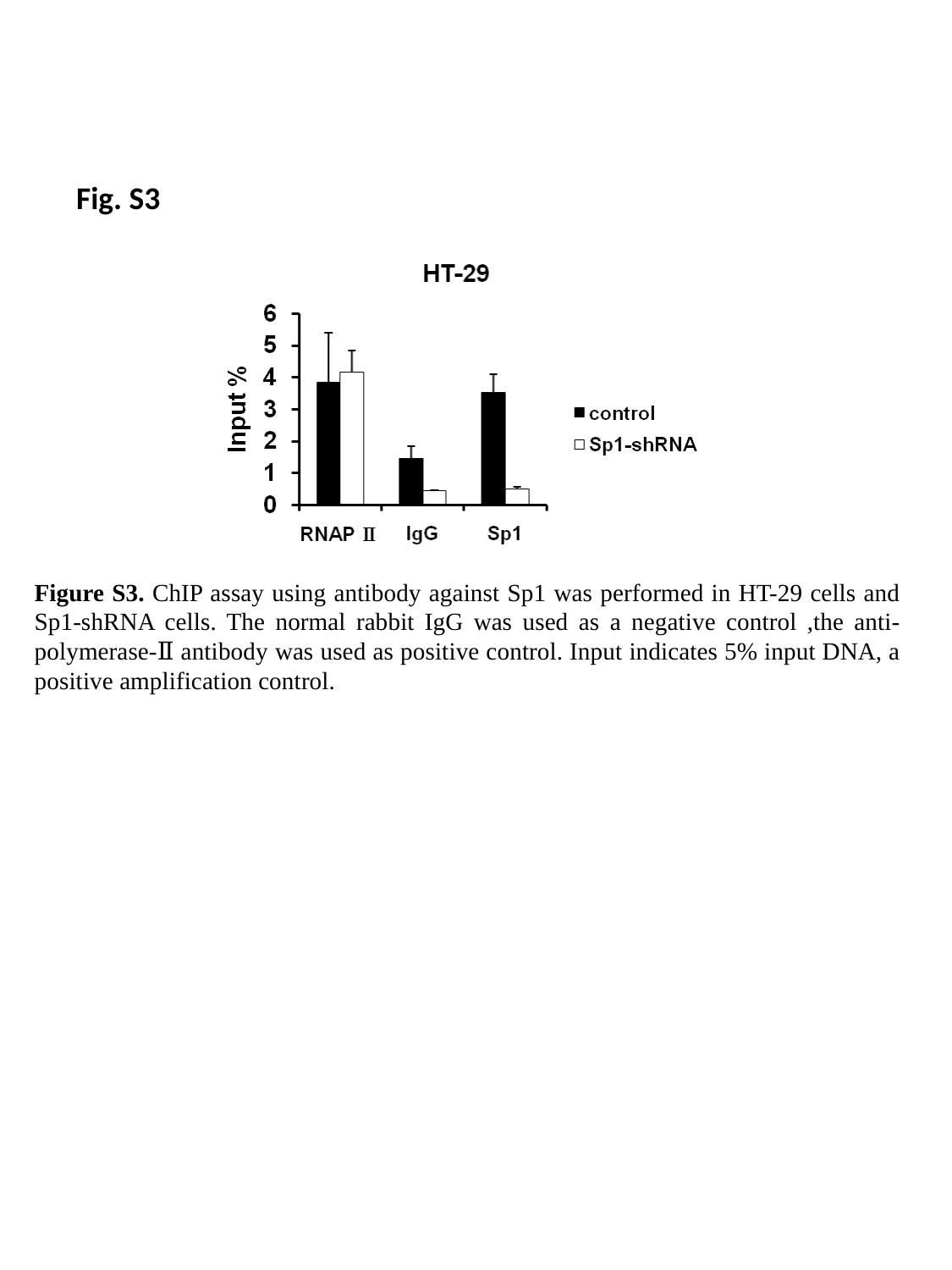

Fig. S3
Figure S3. ChIP assay using antibody against Sp1 was performed in HT-29 cells and Sp1-shRNA cells. The normal rabbit IgG was used as a negative control ,the anti-polymerase-Ⅱ antibody was used as positive control. Input indicates 5% input DNA, a positive amplification control.
