# Supplementary material for: Regulation of RIP3 by the transcription factor Sp1 and the epigenetic regulator UHRF1 modulates cancer cell necroptosis
Source: Cell Death Dis. 2017 Oct 5;8(10):e3084–. doi: 10.1038/cddis.2017.483 (PMC5682651; doi:10.1038/cddis.2017.483)
Supplement: Supplementary Figure S4 [file cddis2017483x4.ppt]

## Slide 1
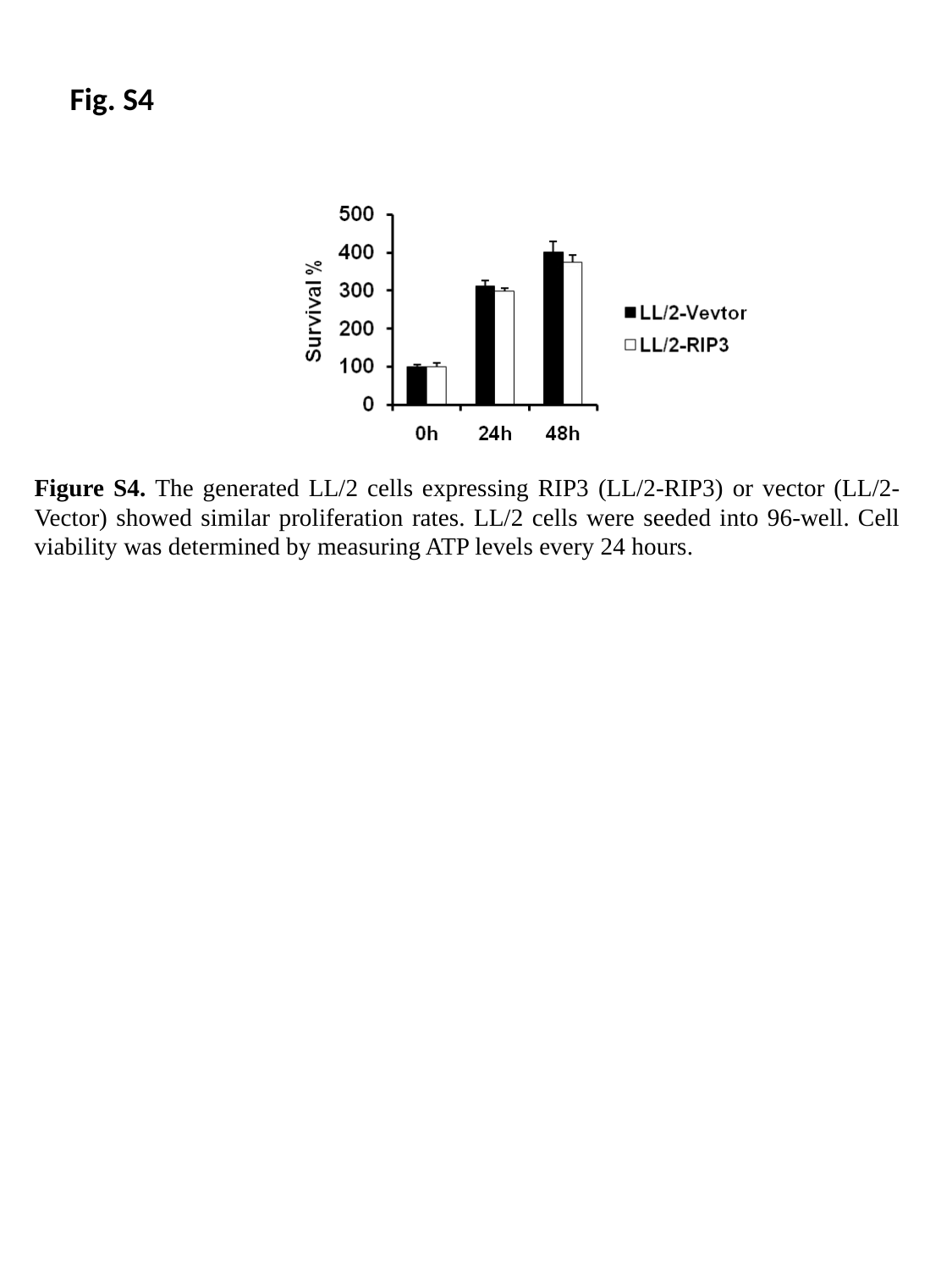

Fig. S4
Figure S4. The generated LL/2 cells expressing RIP3 (LL/2-RIP3) or vector (LL/2-Vector) showed similar proliferation rates. LL/2 cells were seeded into 96-well. Cell viability was determined by measuring ATP levels every 24 hours.
