# Supplementary figures and images for: Regulation of RIP3 by the transcription factor Sp1 and the epigenetic regulator UHRF1 modulates cancer cell necroptosis
Source: Cell Death Dis. 2017 Oct 5;8(10):e3084–. doi: 10.1038/cddis.2017.483 (PMC5682651; doi:10.1038/cddis.2017.483)

## Slide 1
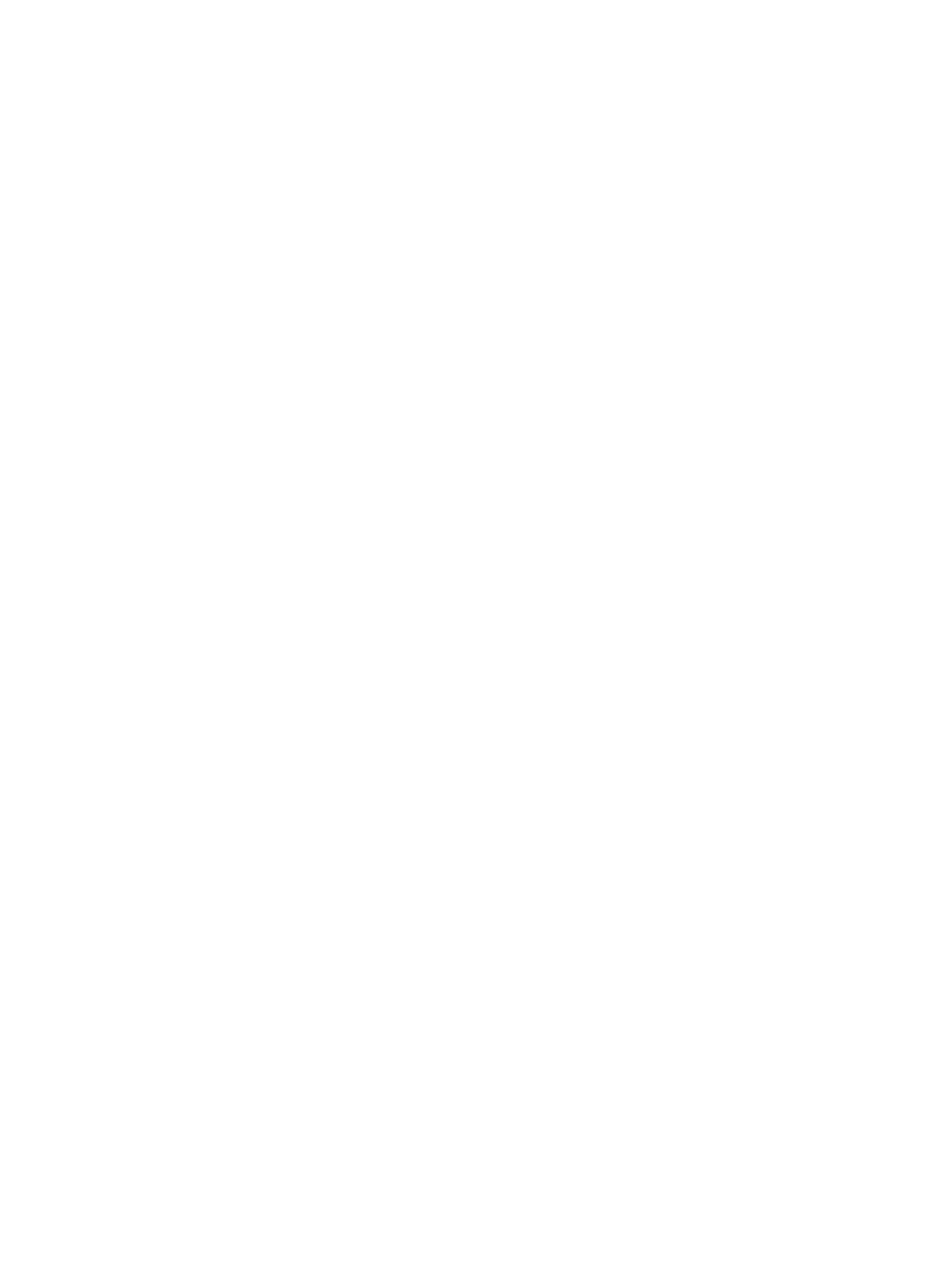

#

Supplement: Supplementary Figure S5 [file cddis2017483x5.ppt]
