# Supplementary material for: Regulation of RIP3 by the transcription factor Sp1 and the epigenetic regulator UHRF1 modulates cancer cell necroptosis
Source: Cell Death Dis. 2017 Oct 5;8(10):e3084–. doi: 10.1038/cddis.2017.483 (PMC5682651; doi:10.1038/cddis.2017.483)
Supplement: Supplementary Figure S6 [file cddis2017483x6.ppt]

## Slide 1
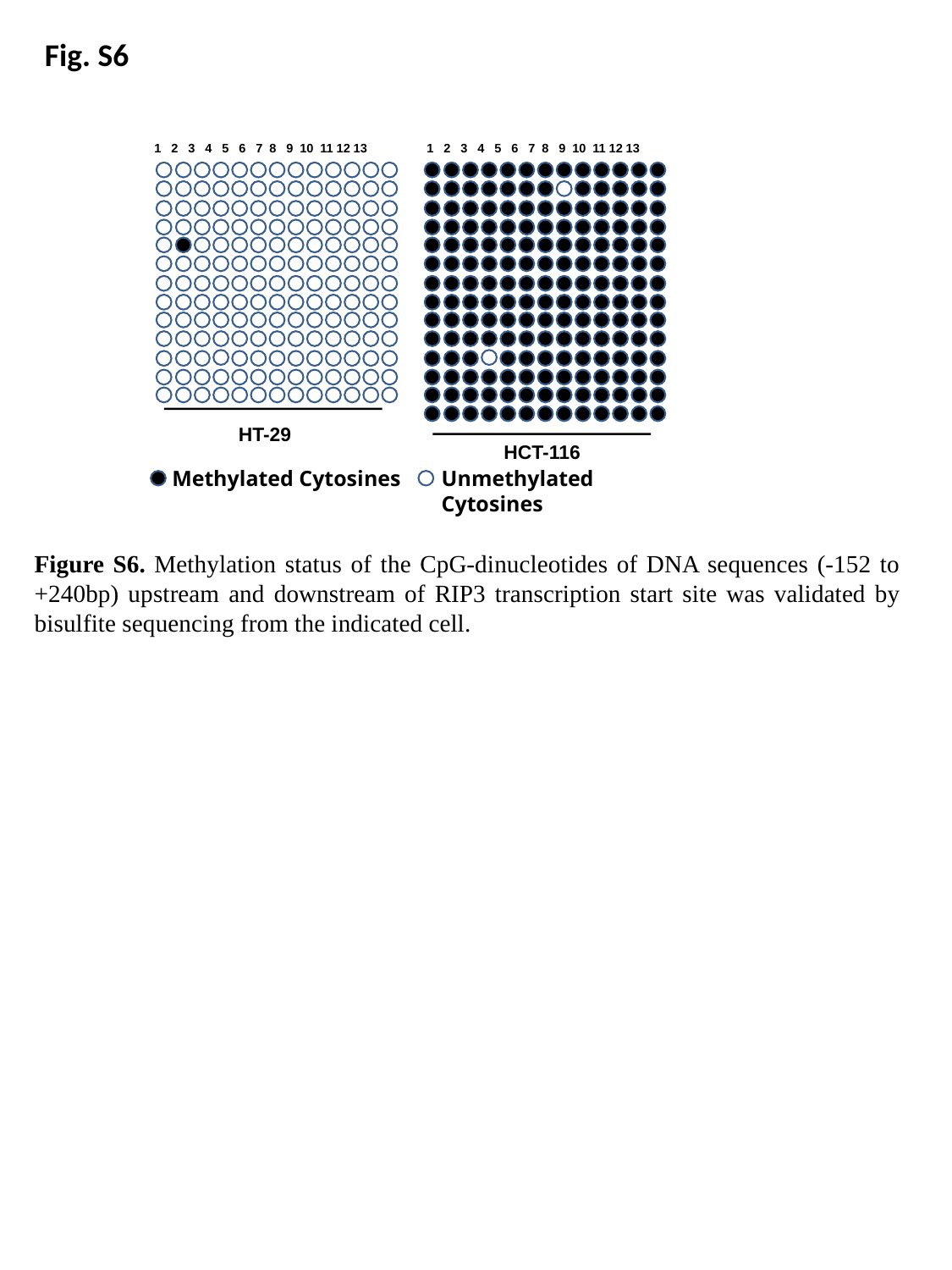

Fig. S6
 1 2 3 4 5 6 7 8 9 10 11 12 13
 1 2 3 4 5 6 7 8 9 10 11 12 13
HT-29
HCT-116
Methylated Cytosines
Unmethylated Cytosines
Figure S6. Methylation status of the CpG-dinucleotides of DNA sequences (-152 to +240bp) upstream and downstream of RIP3 transcription start site was validated by bisulfite sequencing from the indicated cell.
