# Supplementary material for: Regulation of RIP3 by the transcription factor Sp1 and the epigenetic regulator UHRF1 modulates cancer cell necroptosis
Source: Cell Death Dis. 2017 Oct 5;8(10):e3084–. doi: 10.1038/cddis.2017.483 (PMC5682651; doi:10.1038/cddis.2017.483)
Supplement: Supplementary Figure S7 [file cddis2017483x7.ppt]

## Slide 1
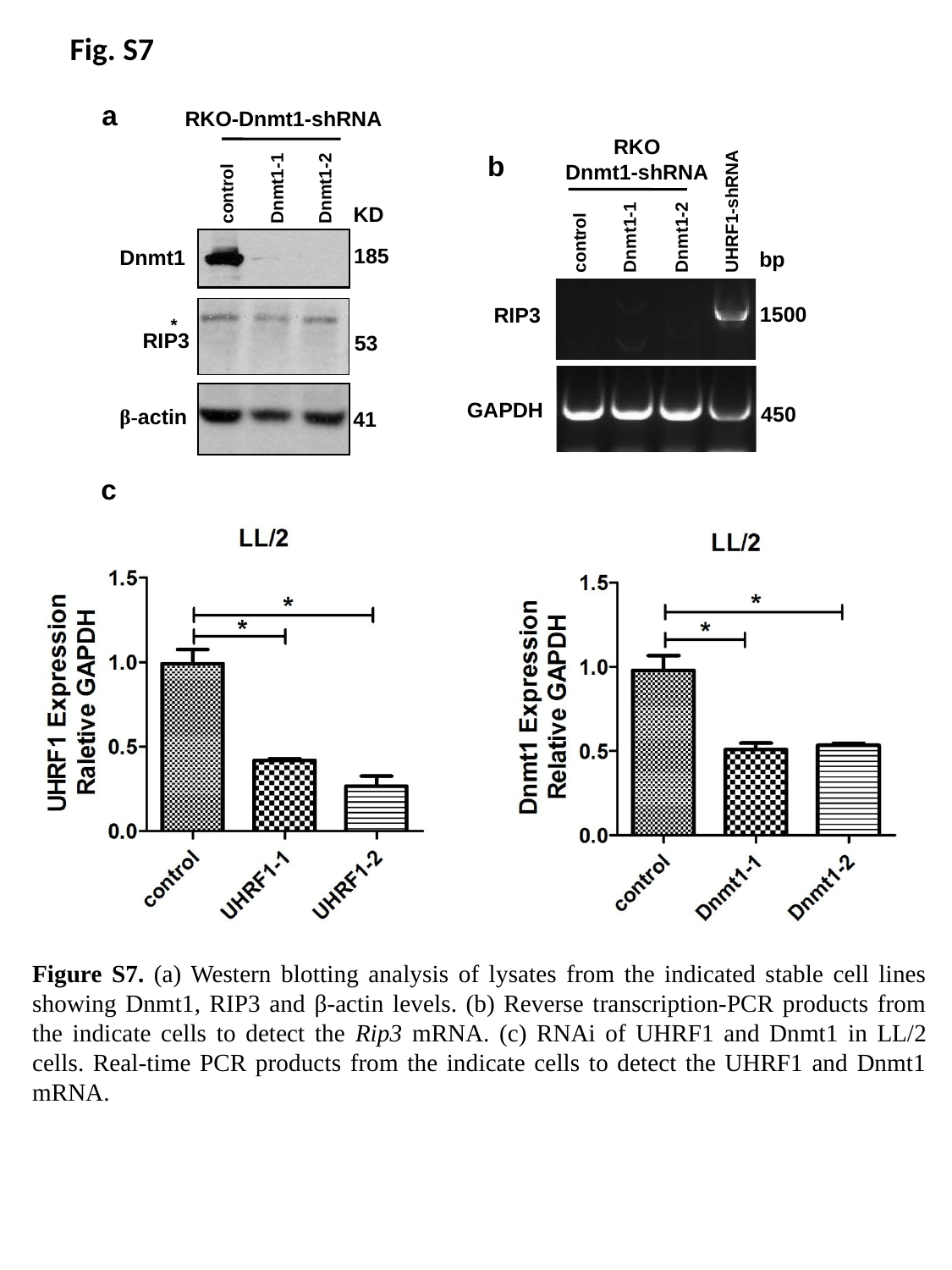

Fig. S7
a
RKO-Dnmt1-shRNA
control
Dnmt1-1
Dnmt1-2
Dnmt1
RIP3
β-actin
*
KD
185
53
41
UHRF1-shRNA
RKO
Dnmt1-shRNA
b
control
Dnmt1-1
Dnmt1-2
RIP3
GAPDH
bp
1500
450
c
Figure S7. (a) Western blotting analysis of lysates from the indicated stable cell lines showing Dnmt1, RIP3 and β-actin levels. (b) Reverse transcription-PCR products from the indicate cells to detect the Rip3 mRNA. (c) RNAi of UHRF1 and Dnmt1 in LL/2 cells. Real-time PCR products from the indicate cells to detect the UHRF1 and Dnmt1 mRNA.
