# Supplementary material for: Regulation of RIP3 by the transcription factor Sp1 and the epigenetic regulator UHRF1 modulates cancer cell necroptosis
Source: Cell Death Dis. 2017 Oct 5;8(10):e3084–. doi: 10.1038/cddis.2017.483 (PMC5682651; doi:10.1038/cddis.2017.483)
Supplement: Supplementary Figure S8 [file cddis2017483x8.ppt]

## Slide 1
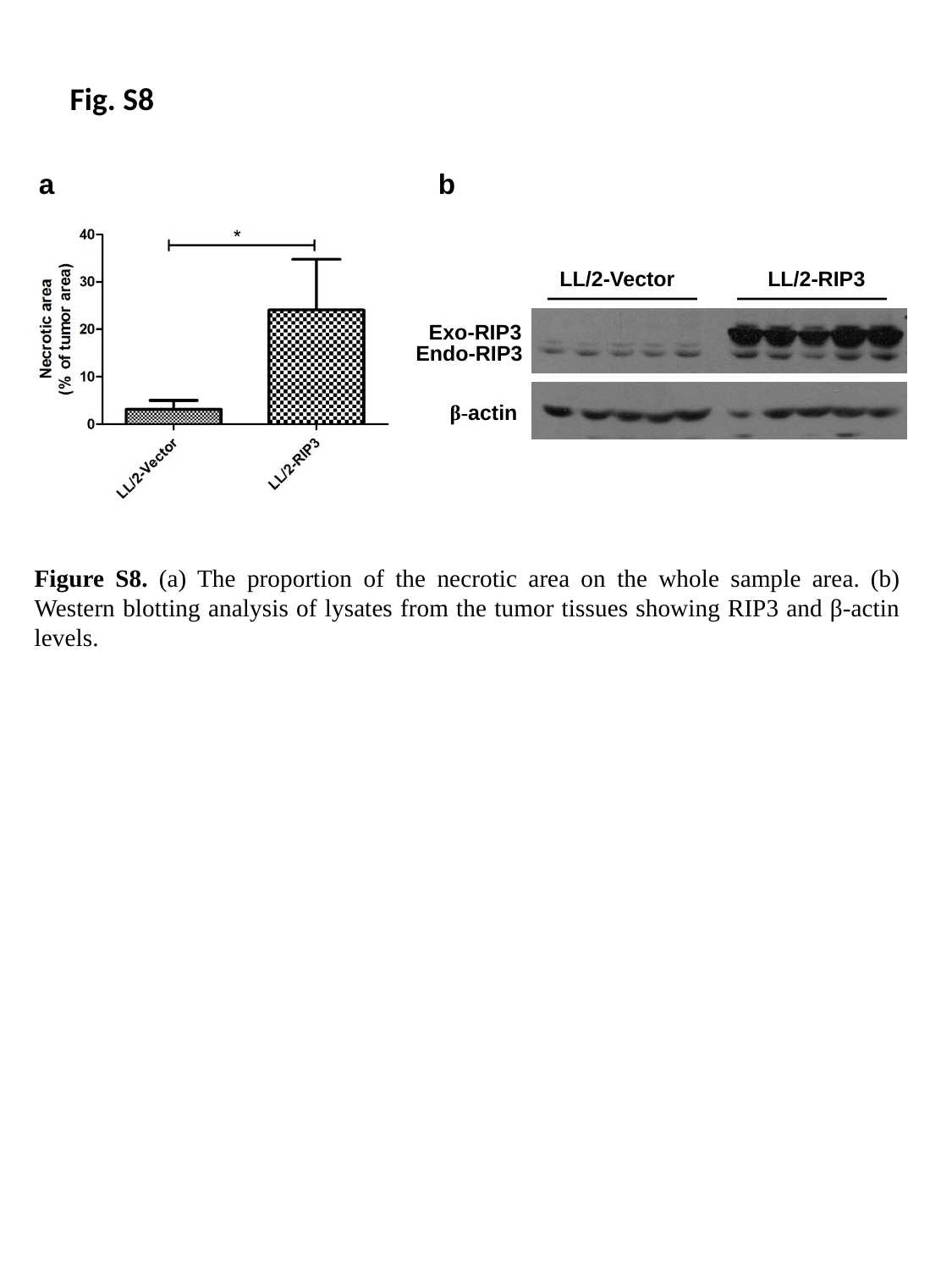

Fig. S8
a
b
LL/2-Vector
LL/2-RIP3
Exo-RIP3
Endo-RIP3
β-actin
Figure S8. (a) The proportion of the necrotic area on the whole sample area. (b) Western blotting analysis of lysates from the tumor tissues showing RIP3 and β-actin levels.
