# Supplementary material for: Regulation of RIP3 by the transcription factor Sp1 and the epigenetic regulator UHRF1 modulates cancer cell necroptosis
Source: Cell Death Dis. 2017 Oct 5;8(10):e3084–. doi: 10.1038/cddis.2017.483 (PMC5682651; doi:10.1038/cddis.2017.483)
Supplement: Supplementary Figure S9 [file cddis2017483x9.ppt]

## Slide 1
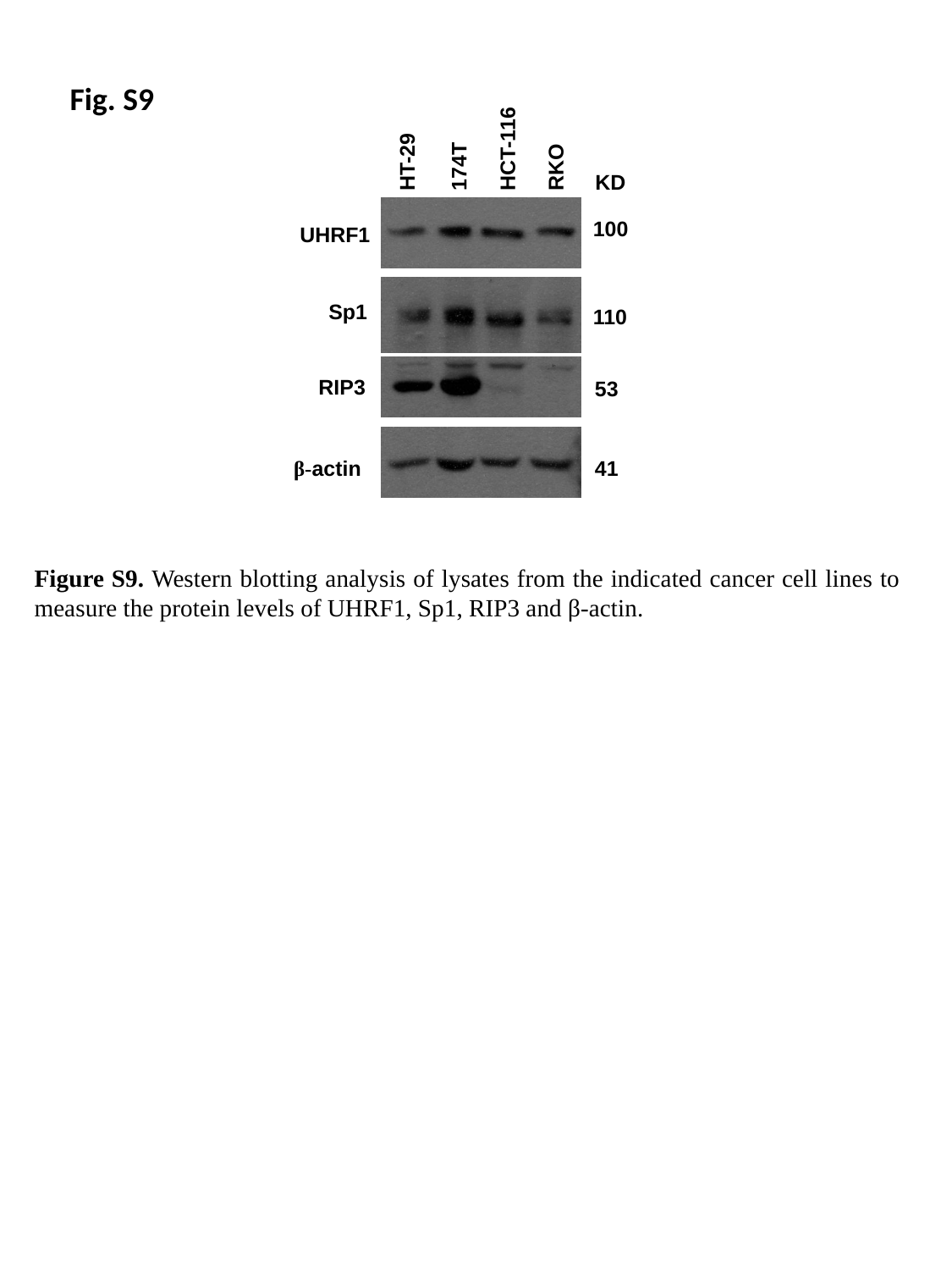

HT-29
174T
HCT-116
RKO
KD
100
UHRF1
Sp1
110
RIP3
53
β-actin
41
Fig. S9
Figure S9. Western blotting analysis of lysates from the indicated cancer cell lines to measure the protein levels of UHRF1, Sp1, RIP3 and β-actin.
